# Supplementary material for: Anatomical and Molecular Characterization of the Zebrafish Meninges
Source: bioRxiv. 2025 Apr 15:2025.04.09.646894. Preprint. [Version 1] doi: 10.1101/2025.04.09.646894 (PMC12047863; doi:10.1101/2025.04.09.646894)
Supplement: Supplement 7 [file NIHPP2025.04.09.646894v1-supplement-7.pdf]

bioRxiv preprint doi: https://doi.org/10.1101/2025.04.09.646894; this version posted April 16, 2025. The copyright holder for this preprint (which was not certified by peer review) is the author/funder. This article is a US Government work. It is not subject to copyright under 17 USC 105 and is also made available for use under a CC0 license.

SUPPLEMENTAL FIGURE LEGENDS

Supplemental Figure 1. Filtering dying cells from the pachymeningeal (dural meningeal) scRNA-seq data set. A, Uniform Manifold Approximation and Projection (UMAP) plots of unfiltered single cell RNA-seq (scRNA-seq) data obtained for dissected pachymeninges (dural meninges), including a total of 12,483 cells. **B,** Heat map of genes enriched in pachymeninges scRNA-seq cell clusters. Clusters 1-12 show similar expression of mitochondrial and ribosomal genes commonly enriched in stressed and/or dying cells. **C,D,** Feature plots of pachymeninges scRNA-seq data showing cells with high levels of mitochondrial (C) or ribosomal (D) gene expression. **E,** Feature plot highlighting cells that showed enriched mitochondrial and ribosomal gene expression and limited cluster-specific gene expression (red). All of these cell clusters (8,346 cells in total) were removed from the final filtered pachymeningeal UMAP showed in Figure 3B.

Supplemental Figure 2. Filtering neural cells from the leptomeningeal scRNA-seq data set. A, UMAP plot of unfiltered single cell RNA-seq (scRNA-seq) data obtained for dissected leptomeninges, including a total of 22,587 cells. **B,** Feature plot showing leptomeningeal scRNA-seq cell clusters with a mature or precursor neuronal cell identity based on their gene expression profile (red). Individual feature plots show the distribution of mature neuron markers expressed by these cells (*gap43, eno2, map2, nefla, uchl1, rbfox3a*). All of these cell clusters 17,109 cells in total) were removed from the final filtered leptomeningeal UMAP showed in Figure 3C. **C,D,** Feature plots highlighting cells in red expressing markers consistent with a GABAergic (C) or glutamatergic (D) neuronal identity.

Supplemental Figure 3. Habenular neurons in the leptomeningeal scRNA-seq data set. A, Feature plots highlighting leptomeningeal scRNA-seq cell clusters (red) containing cells with medial habenula (MHb, left) or lateral habenula (LHb, right) identity. **B,** Dot plot showing genes expressed by leptomeningeal scRNA-seq cell clusters including putative MHb and LHb clusters (highlighted by light red bars). The MHb shows almost no expression of *gap43* and lower expression of *snap25b*, while the LHb shows characteristic high expression of *gap43* and *snap25b*.

Supplemental Figure 4. Vascular and vascular-associated cells in the leptomeninges. A, Dorsal view of a *casper* adult zebrafish head. The asterisk notes the optic tectum lobe area where the images in B-C were collected. **B-G,** Confocal images of EGFP-positive pericytes (B,D,E,G; green) and mCherry-positive blood vessel endothelial cells (B,C,E,F; magenta) in the leptomeninges of a dissected brain from a *Tg(kdrl:mcherry)*^{y206}, *Tg(pdgfrb:egfp)*^{ncv22} double transgenic adult zebrafish. Panels E-G show higher magnification images of blood vessels with pericytes. **H-M,** Confocal images of EGFP-positive FGPs (H,J,K,M; green) and mCherry-positive blood vessel endothelial cells (H,I,K,L; magenta) in the leptomeninges of a dissected brain from a *Tg(kdrl:mcherry)*^{y206}*, Tg(mrc1a:egfp)*^{y251} *double transgenic adult zebrafish* double transgenic adult zebrafish. Panels K-M show higher magnification images of blood vessels with FGPs. Scale bars = 200 µm (B-D), 20 µm (E-G), 50 µm (H-J), and 10 µm (K-M).

Supplemental Figure 5. Identification of erythrocytes in meningeal scRNA-seq data sets. A, Pachymeningeal feature plots showing adult hemoglobins expressed in the pachymeningeal scRNA-seq data set, with very strong expression in the erythrocyte cluster. **B,** Leptomeningeal

bioRxiv preprint doi: https://doi.org/10.1101/2025.04.09.646894; this version posted April 16, 2025. The copyright holder for this preprint (which was not certified by peer review) is the author/funder. This article is a US Government work. It is not subject to copyright under 17 USC 105 and is also made available for use under a CC0 license.

feature plots showing adult hemoglobins expressed in the leptomeningeal scRNA-seq data set, with very strong expression in the erythrocyte cluster.

Supplemental Figure 6. Meningeal precursor, meningeal fibroblast, and LBC clusters in the Daniocell mesenchyme scRNA-seq data set. A,D,G, UMAP plots of the mesenchymal subset of the Daniocell (https://daniocell.nichd.nih.gov/) scRNA-seq data set, highlighting the cells in each of the three clusters. **B,E,H,** Graphical plot of the normalized percentage of cells present at each developmental stage of the Daniocell data set, for each of the three clusters. **C,F,I,** Graphical plot of the number of cycling vs. non- cycling cells present at each developmental stage of the Daniocell data set, for each of the three clusters. Clusters shown in the panels are Mese.21 "Meninges Precursors" (**A-C**), Mese.29 "Meninges Leptomeninges" (**D-F**; e.g., LBCs), or Mese.32 "Meningeal Fibroblasts" (**G-I**). Mese.21 Meninges Precursors are situated at the base of both Mese.29 Meninges Leptomeninges and Mese.32 Meningeal Fibroblast cell clusters (A,D,G), and they appear at earlier stages than LBCs and meningeal fibroblasts (B,E,H), and include many more cycling cells, especially at the earlier developmental stages (C,F,I).

Supplemental Figure 7. Supplementary data for LBCs vs. meningeal fibroblasts. A, Dot plot showing enriched expression of selected diagnostic genes in meningeal fibroblasts, LBCs, or both in the mesenchymal subset of the Daniocell scRNA-seq data set. Average expression and percent of cells in each cluster expressing each gene are indicated by dot color and size, respectively, with average expression on a log2 scale. **B,** Pseudotime plot of cells in the Mese.21 "Meninges Precursor," Mese.29 "Meninges Leptomeninges," and Mese.32 "Meningeal Fibroblast" clusters, with color coding showing the actual developmental stages of each cell. **C,** Pseudotime plots of cells in the Mese.21 "Meninges Precursor," Mese.29 "Meninges Leptomeninges," and Mese.32 "Meningeal Fibroblast" clusters, showing expression of genes enriched in either LBCs (top row) or meningeal fibroblasts (bottom row).

Supplemental Figure 8. Apoptotic LBC cells are phagocytosed by adjacent FGPs after LBC ablation in the adult leptomeninges. A-F. Confocal micrographs of transgenic *Tg(epd:gal4-VP16);Tg(uas:ntr-mcherry);Tg(mrc1a:egfp)* adult animals treated with either DMSO (A-C) or 10mM MTZ (D-F). Top panels show a control DMSO treated animal with intact LBC (magenta) and FGP (green) leptomeningeal coverage. Bottom panels (D-F) show an animal with ablated LBCs, where FGPs (green) have engulfed the LBC-resulting debris (magenta) showing strong magenta inclusions inside them.

SUPPLEMENTAL MOVIE LEGENDS

Supplemental Movie 1. T cells, lymphatics, and FGPs in the meninges. 3-D reconstructed views of a confocal Z-stack imaged through the skull of an intact, living *casper, Tg(lck:mcherry)^{ns107}*, *Tg(mrc1a:eGFP)^{y251}* double transgenic adult zebrafish, showing T cells (magenta) in and around lymphatic vessels (green) in the dural meninges to FGPs in the leptomeninges. Blue autofluorescence is used to visualize the skull located immediately above the meningeal layers.

Supplemental Movie 2. LBCs on the surface of the larval zebrafish brain. The first half of the movie shows a 14 hour time-lapse confocal imaging of LBC nuclei (magenta) and cell bodies (green) over the surface of an approximately 2-2.5 dpf *Tg(epd:mcherry)^{y715}*, *Tg(epd:gfp-caax)^{y716}*

bioRxiv preprint doi: https://doi.org/10.1101/2025.04.09.646894; this version posted April 16, 2025. The copyright holder for this preprint (which was not certified by peer review) is the author/funder. This article is a US Government work. It is not subject to copyright under 17 USC 105 and is also made available for use under a CC0 license.

larval brain (selected frames shown in **Fig. 6H-L**). Yellow arrows show dividing LBCs. The second half of the movie shows rotating 3D reconstruction views of a confocal stack of LBCs on a 5 dpf *Tg(epd:gfp-caax)*^{y716} brain, revealing that LBCs are located only on the surface of the brain (sequence begins with anterior to the left, dorsal in foreground).

Supplemental Movie 3. The adult zebrafish leptomeninges is composed largely of LBCs. 3-D reconstructed views of a confocal Z-stack through the leptomeninges on the surface of a dissected *Tg(epd:mCherry), Tg(mrc1a:eGFP)* double transgenic adult zebrafish brain, with LBCs in magenta and FGPs in green. Blood vessels are unlabeled but appear as black tubular structures surrounded by LBCs and FGPs. Movie begins with a dorsal (X-Y) view then rotates to show lateral (X-Z) slices with blood vessels and FGPs running through a continuous LBC layer. Scale bar = 25mm. Selected frames shown in **Fig. 6P,Q**).

Supplemental Movie 4. Metronidazole-dependent LBC ablation in larval zebrafish. Video showing lateral views of 5 dpf transgenic *Tg(epd:gal4-VP16);Tg(uas:ntr-mcherry);Tg(mrc1a:egfp)* zebrafish larvae treated with either 10mM MTZ (upper panel) or DMSO (lower panel) showing MTZ-NTR dependent induction of LBC apoptosis. Time lapse was started at 5 dpf with acquisition intervals every 10 minutes for a total of 12 hours.

Supplemental Movie 5. Control and LBC-ablated adult zebrafish. Video images of the gross behavioral effects of acute LBC ablation on adult zebrafish. Control DMSO carrier only treated (LBCs intact) animals are in the lower tank, and 10mM MTZ treated (LBCs ablated) animals are in the upper tank. Animals are shown 12 hours post-initiation of treatment (comparable to the animals shown in **Fig. 8O-V**). Control animals are unaffected, but ablated animals are unresponsive and swimming slowly or unable to swim. Most EDP-ablated animals are still alive (possessing an active heartbeat) at this stage, but all will die within hours.

Supplemental Movie 6. Acute ablation of LBC cells in adult animals. Video shows 3D renderings of confocal micrographs of either control (left) or MTZ-treated adult *Tg(epd:gal4-VP16);Tg(uas:ntr-mcherry);Tg(mrc1a:egfp)* transgenic animals following exposure to DMSO or 10mM MTZ for 24 hours. Images were obtained on alive animals through intact skulls to preserve meningeal anatomy. LBCs are shown in magenta, FGPs and lymphatics are in green and skull (autofluorescence) is shown in blue. MTZ-treated animal shows apoptotic LBCs (magenta) being phagocytosed by FGPs (green).

SUPPLEMENTAL FILES

Supp Figs S1-S8: Venero Galanternik et al – Supp Figures 1-8.pdf
Supplemental Movie 1. Venero_et_al_Supp_Movie_1.mp4
Supplemental Movie 2. Venero_et_al_Supp_Movie_2.mp4
Supplemental Movie 3. Venero_et_al_Supp_Movie_3.mp4
Supplemental Movie 4. Venero_et_al_Supp_Movie_4.mp4
Supplemental Movie 5. Venero_et_al_Supp_Movie_5.mp4
Supplemental Movie 6. Venero_et_al_Supp_Movie_6.mp4

SUPPLEMENTAL FIGURE 1

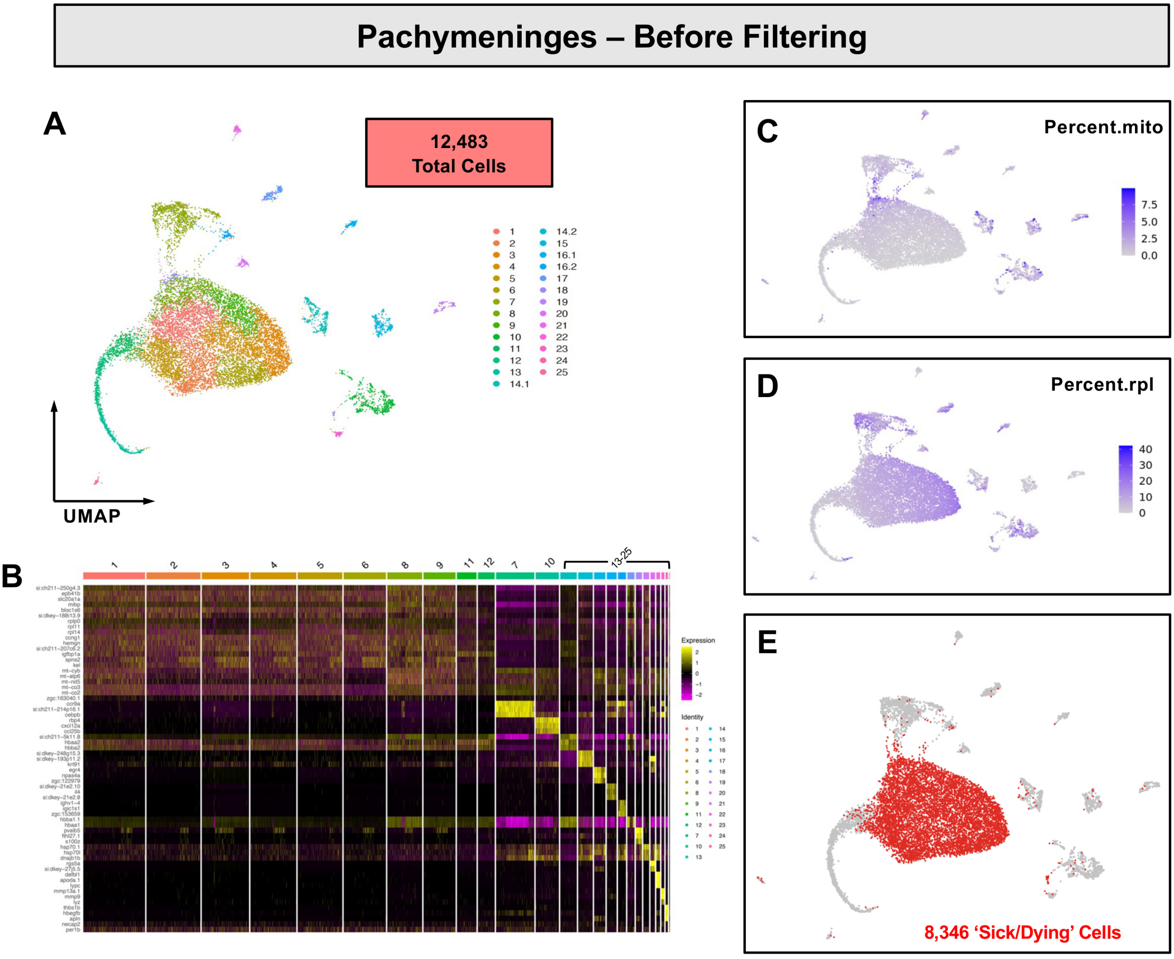

SUPPLEMENTAL FIGURE 2

Leptomeninges – Before Filtering

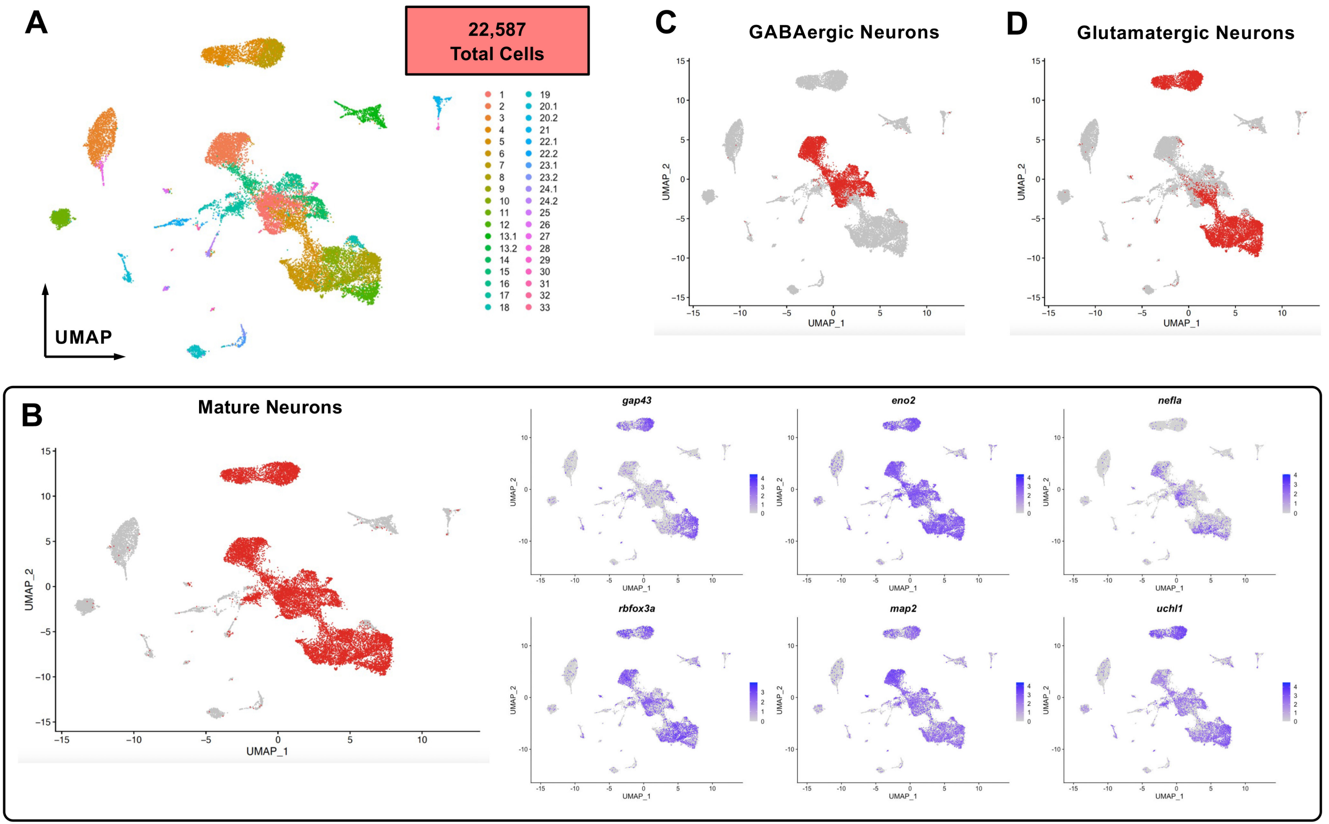

SUPPLEMENTAL FIGURE 3

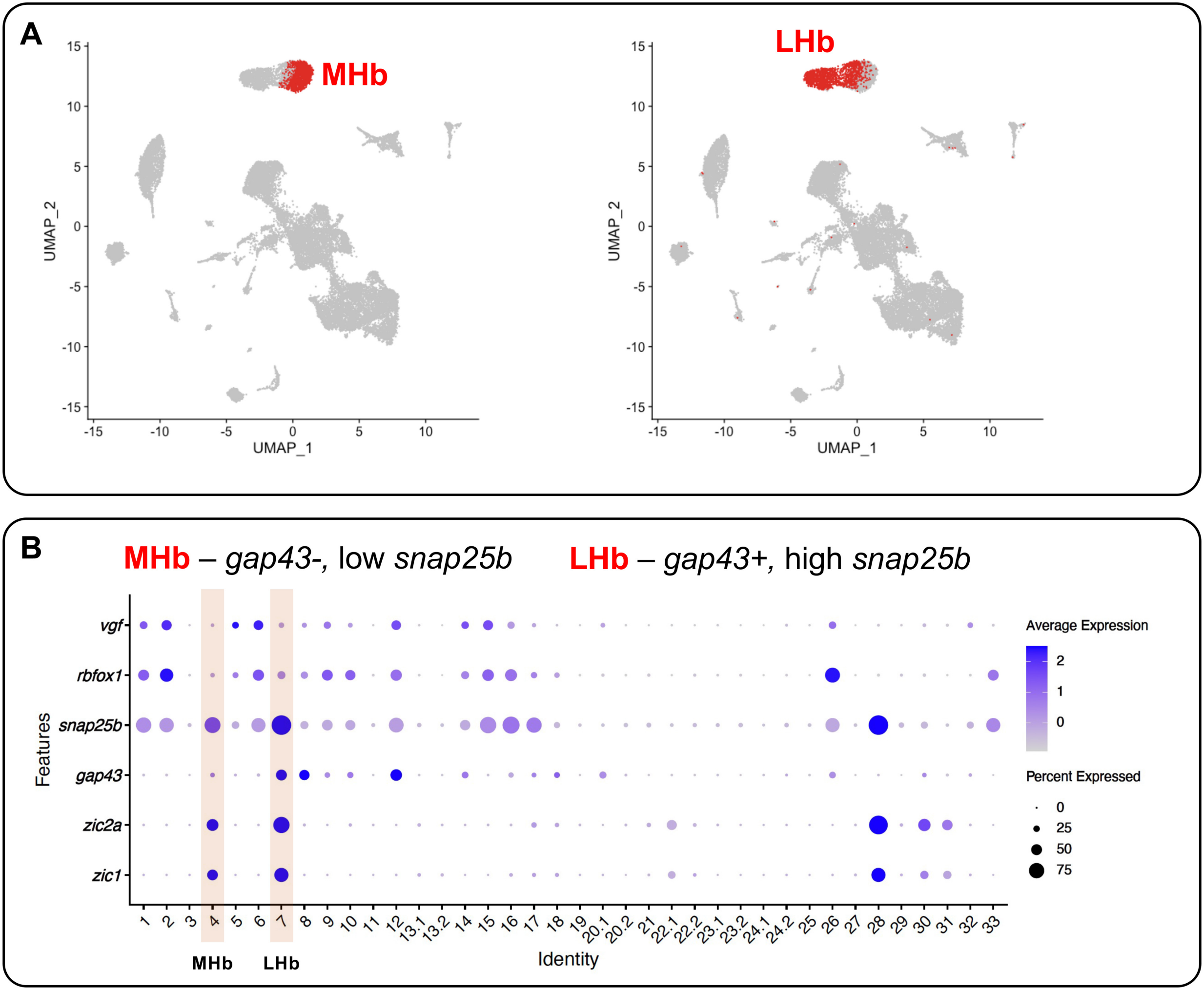

SUPPLEMENTAL FIGURE 4

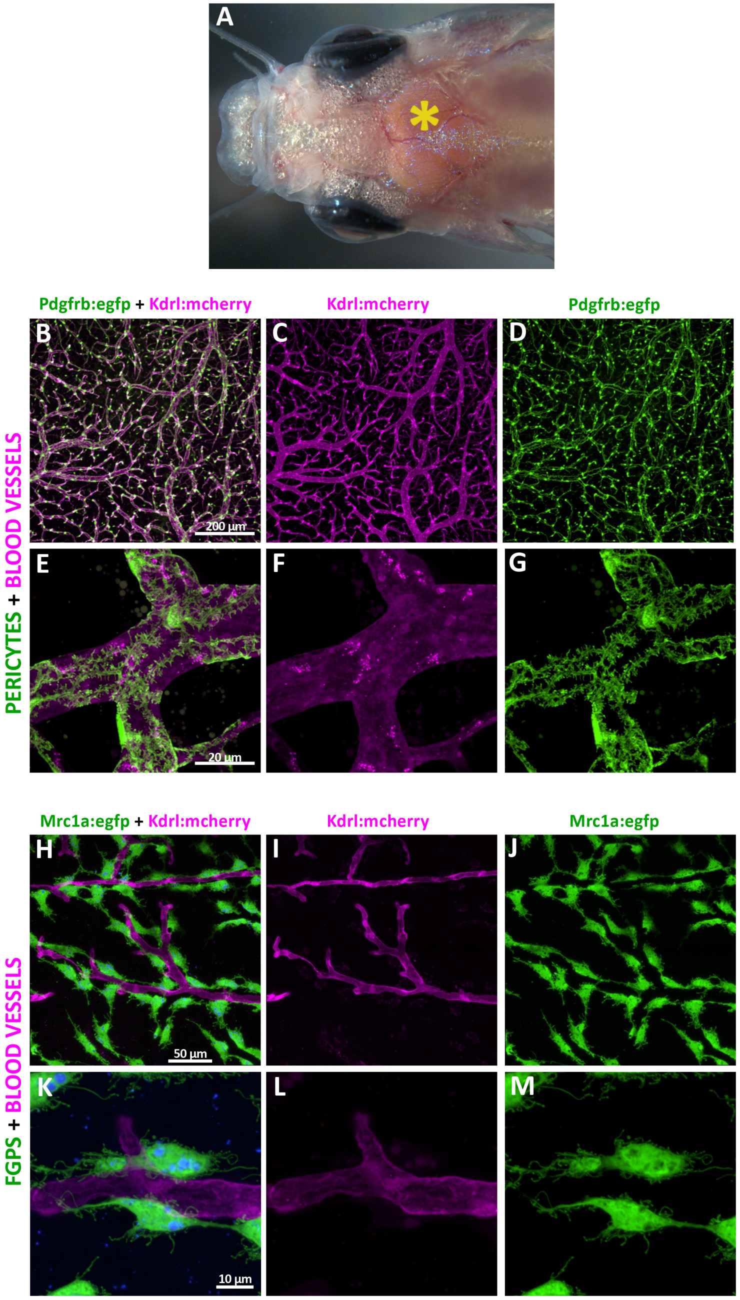

SUPPLEMENTAL FIGURE 5

Pachymeninges

A

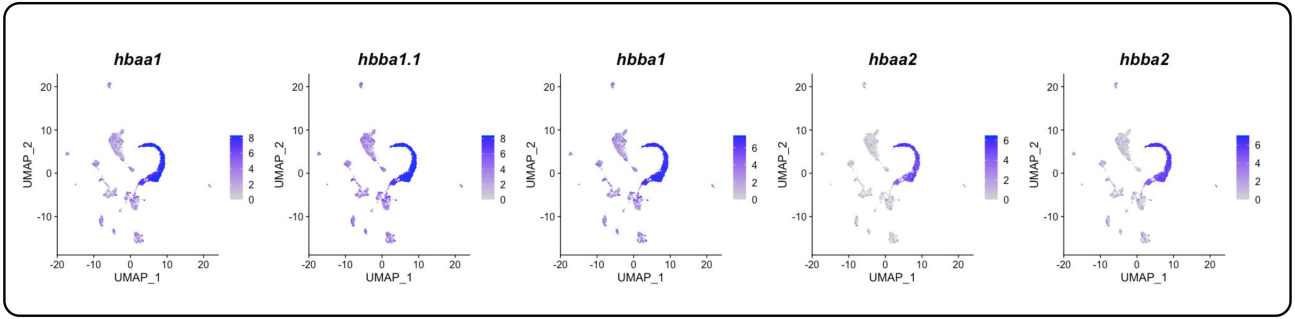

Leptomeninges

B

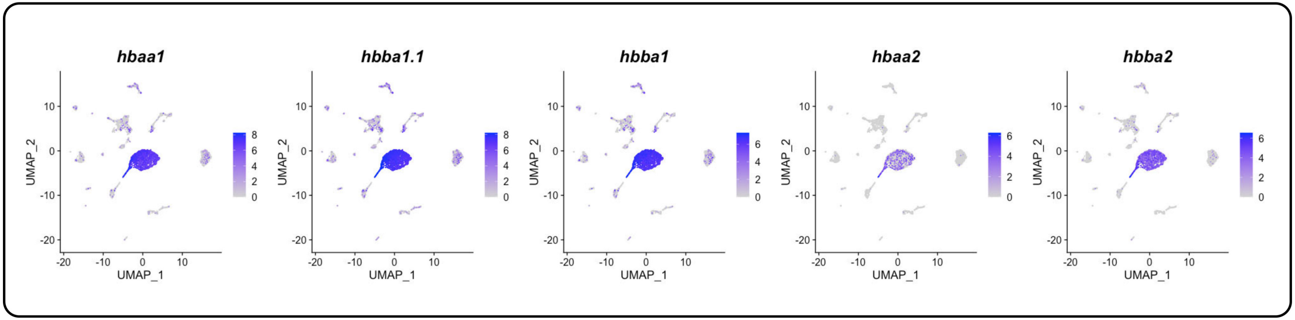

SUPPLEMENTAL FIGURE 6

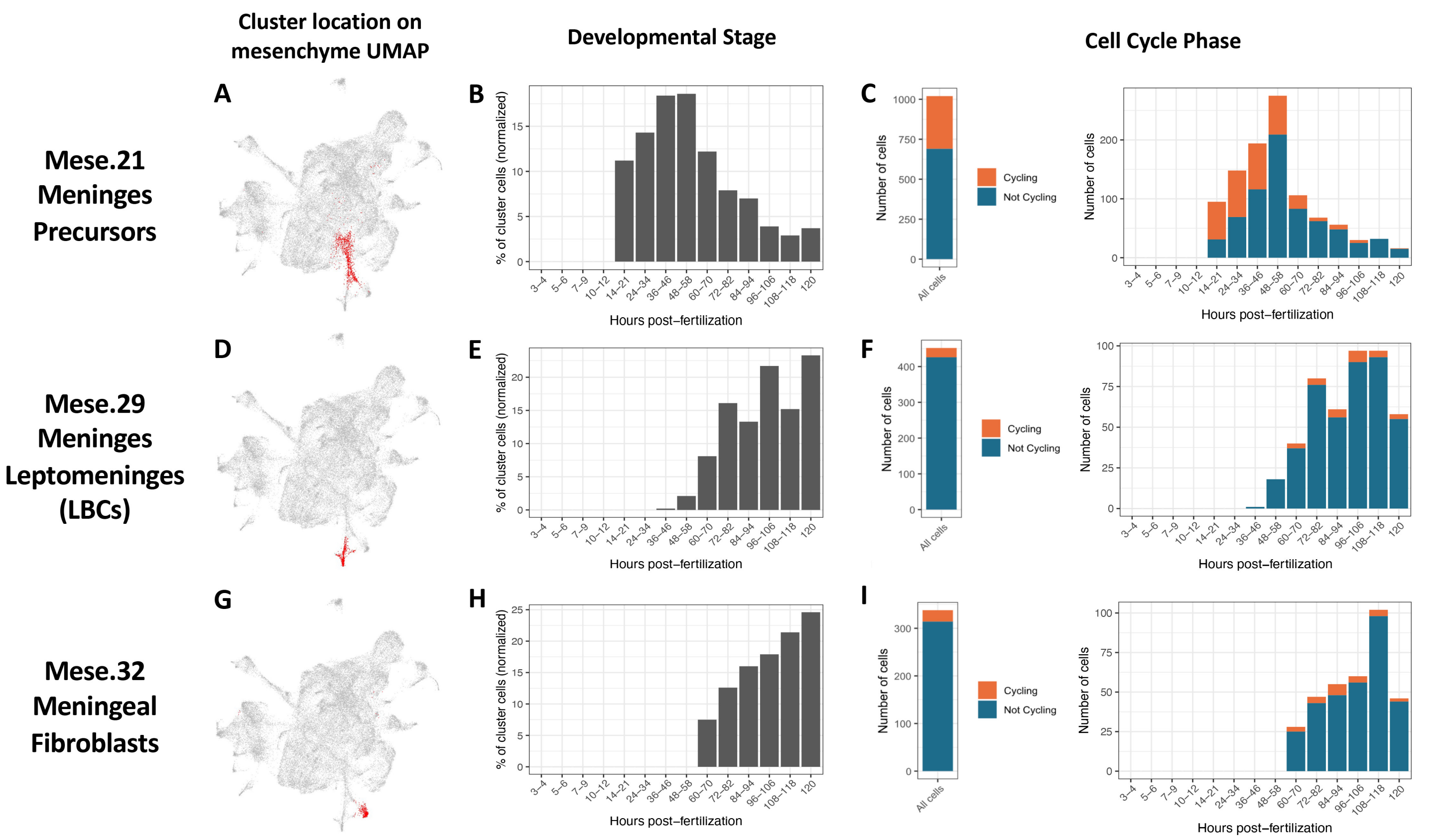

SUPPLEMENTAL FIGURE 7

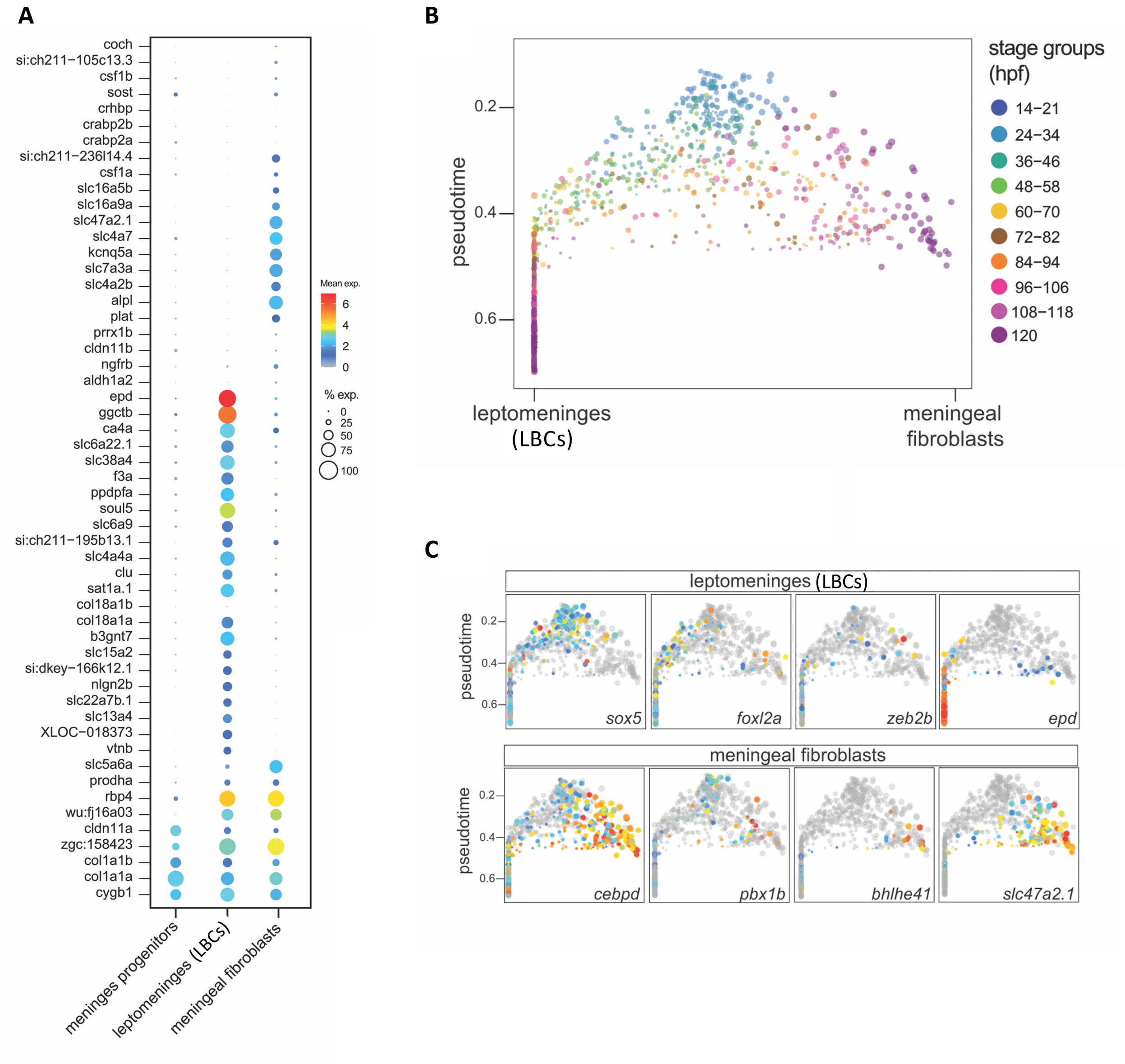

SUPPLEMENTAL FIGURE 8

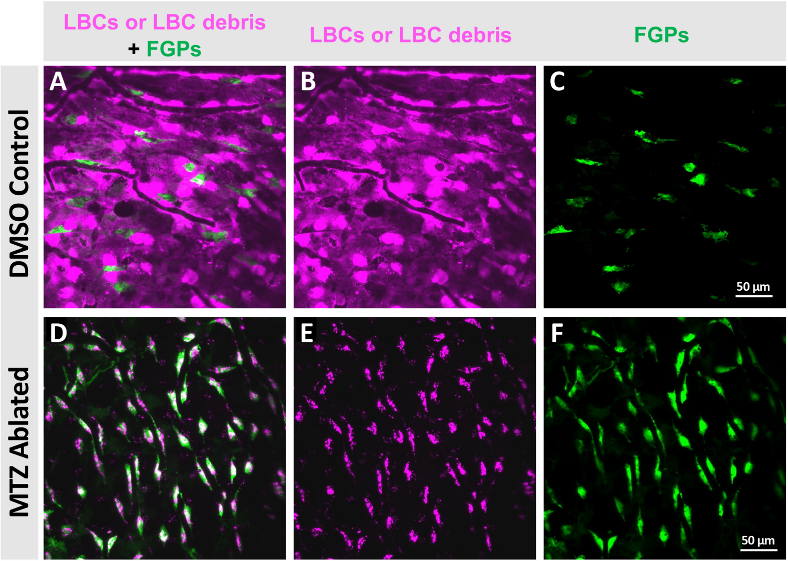

SUPPLEMENTAL TABLE 2

Pachymeninges

Cell Type	Gene Name			
T Lymphocyte	*sla2*	*il2rb*	*tcf7*	*tnfsf9b*
Macrophage	*mpeg1.1*	*c1qc*	*c1qb*	*grn1*
B Lymphocyte	*igl1c3*	*igl3v5*	*igic1s1*	*cd37*
Endothelial	*kdrl*	*kdr*	*egfl7*	*cldn5b*
Glia	*gfap*	*s100b*	*slc1a2b*	*cx43*
GABAergic Neuron	*gad1b*	*gad2*	*gpm6aa*	*nrxn3a*
Glutamatergic Neuron	*slc17a6a*	*grin1a*	*grin1b*	*grin2bb*
Neutrophil	*mmp9*	*mpx*	*lyz*	*mmp13a*
Thrombocyte	*fn1b*	*f5*	*thbs1b*	*apln*
Erythrocyte	*hbba1*	*hbba1.1*	*hbaa1*	*hbba2*
Pigment	*pnp4a*	*gpnmb*	*tfec*	*sox10*
Periderm	*cldne*	*epcam*	*cd9b*	*ftr83*
Mural	*notch3*	*pdgfrb*	*myh11a*	*fgfbp2a*
Fibroblast	*pdgfra*	*slc4a7*	*pdgfrb*	*slc16a9a*
Epithelial	*vil1*	*krt4*	*tuft1a*	*egr3*
Myeloid Precursor	*cd74a*	*cd9b*	*cd74b*	*si:dkey-83f18.9*

Leptomeninges

Cell Type	Gene Name			
T Lymphocyte	*sla2*	*il2rb*	*tcf7*	*tnfrsf9b*
Macrophage	*mpeg1.1*	*cmklr1*	*csf1ra*	*havcr1*
B Lymphocyte	*igl1c3*	*igl3v5*	*igic1s1*	*cd37*
Endothelial	*kdrl*	*cdh5*	*cldn5a*	*flt1*
Glia	*s100b*	*slc1a2b*	*aqp1a.1*	*cx43*
Oligodendrocyte	*mbpa*	*rtn4a*	*plp1b*	*olig2*
Oligodendrocyte Precursor	*cspg4*	*sox10*	*olig1*	*aplnra*
GABAergic Neuron	*gabrb3*	*pvalb6*	*calb2a*	*gad2*
Glutamatergic Neuron	*slc17a6a*	*grin1a*	*slc17a6b*	*grin2bb*
Dendritic	*irx1b*	*ccl44*	*gnai3*	*inhbab*
FGP	*mrc1a*	*lyve1b*	*flt4*	*loxl1*
Fibroblast	*slc47a2.1*	*slc4a7*	*slc16a9a*	*slc16a5b*
LBC	*epd*	*ggctb*	*slc13a4*	*cp*
Erythrocyte	*hbba1*	*hbba1.1*	*hbaa1*	*hbba2*
Mural	*notch3*	*pdgfrb*	*myh11a*	*rgs5a*
Epithelial	*prom2*	*vcanb*	*smoc2*	*erbb3a*
Myeloid	*ccl34b.1*	*cd74a*	*cd74b*	*mhc2dab*
